# An Updated Review of Resistin and Colorectal Cancer

**DOI:** 10.7759/cureus.65403

**Published:** 2024-07-26

**Authors:** Aliki Vaia Rompou, Garyfalia Bletsa, Dimitris Tsakogiannis, Stamatios Theocharis, Panteleimon Vassiliu, Nick Danias

**Affiliations:** 1 Department of Colorectal Surgery, Guy's and St Thomas' NHS Foundation Trust, London, GBR; 2 Department of Medicine, Research Center, Hellenic Anticancer Institute, Athens, GRC; 3 Department of Pathology, National and Kapodistrian University of Athens, Athens, GRC; 4 Fourth Department of Surgery, Attikon University Hospital, National and Kapodistrian University of Athens, Athens, GRC

**Keywords:** adipocytokine, biochemistry, molecular biology, clinical biochemistry, oxidative stress, cancer research, obesity and cancer, obesity, colorectal cancer, resistin and cancer

## Abstract

Resistin is one of the most important adipokines, and its role lies mainly in controlling insulin sensitivity and inflammation. However, over the last years, the study of resistin gained increased popularity since it was proved that there is a considerable relationship between high levels of resistin and obesity as well as obesity-induced diseases, including diabetes, cardiovascular disorders, and cancer. Regarding cancer risk, circulating resistin levels have been correlated with several types of cancer, including colorectal, breast, lung, endometrial, gastroesophageal, prostate, renal, and pancreatic cancer.

Colorectal cancer is regarded as a multi-pathway disease. Several pathophysiological features seem to promote colorectal cancer (CRC) such as chronic inflammation, insulin resistance, and obesity. Even though the molecular mechanisms involved in CRC development remain rather vague, it is widely accepted that several biochemical factors promote CRC by releasing augmented pro-inflammatory cytokines, like IGF-I, insulin, sex-steroid hormones, and adipokines. A wide range of research studies has focused on evaluating the impact of circulating resistin levels on CRC risk and determining the efficacy of chemotherapy in CRC patients by measuring resistin levels. Moreover, significant outcomes have emerged regarding the association of specific single nucleotide polymorphisms (SNPs) in the resistin gene and CRC risk.

The present study reviewed the role of circulating resistin levels in CRC development and shed light on specific resistin gene SNPs implicated in the disease's development. Finally, we analyzed the impact of resistin levels on the effectiveness of chemotherapy and further discussed whether resistin can be regarded as a valuable biomarker for CRC prognosis and treatment.

Resistin is one of the most important adipokines, and its role lies mainly in controlling insulin sensitivity and inflammation. However, over the last years, the study of resistin gained increased popularity since it was proved that there is a considerable relationship between high levels of resistin and obesity as well as obesity-induced diseases, including diabetes, cardiovascular disorders, and cancer. This review discusses the aberrant expression of resistin and its receptors, its diverse downstream signaling, and its impact on tumor growth, metastasis, angiogenesis, and therapy resistance to support its clinical exploitation in biomarker and therapeutic development.

## Introduction and background

Resistin is an adipokine that was originally discovered in 2001 by Dr. Mitchell Lazar's group as a link between diabetes and obesity, since then, it has emerged as a potential player in the pathogenesis of colorectal cancer (CRC) [1]. While resistin was initially thought to play a fundamental role in regulating insulin resistance and inflammation, recent studies have shown a significant association between high levels of resistin and obesity-related diseases, including diabetes, cardiovascular disease, and cancer [1,2]. Circulating resistin concentrations have been associated with several types of cancer, including colorectal, breast, lung, uterine, gastrointestinal, prostate, kidney, and pancreatic cancer [3-12].

CRC is one of the most commonly diagnosed cancers worldwide. Traditionally, the mechanisms of CRC development have focused on genetic alterations, including chromosomal damage and microsatellite instability [13]. However, recent studies have shown that pathophysiological features such as chronic inflammation, insulin resistance, and obesity also play an important role in the development of CRC [14]. Biochemical factors, including pro-inflammatory cytokines such as IGF-I, insulin, sex steroid hormones, and adipokines such as resistin, are thought to promote the development of CRC [15-19].

Numerous research studies have focused on investigating the effects of circulating resistin levels on CRC risk, progression, metastasis, invasion, tumor angiogenesis, and chemotherapy efficacy in CRC patients [20-22]. In addition, specific single nucleotide polymorphisms (SNPs) in the resistin gene have been associated with CRC risk [23]. Consequently, targeting resistin and its downstream signaling pathways may provide new therapeutic opportunities for the treatment of CRC. Further studies are needed to unravel the precise mechanisms underlying the role of resistin in CRC and explore its potential as a diagnostic marker and therapeutic target. This article reviews the potential role of resistin in the pathogenesis of CRC and its impact on tumor progression.

Resistin was named for its ability to resist the action of insulin and has been proposed as a link between obesity and diabetes [24]. It belongs to a family of resistin-like molecules (RLMs) with different expression patterns and biological effects [1,25]. Resistin is expressed in various cell types, including adipocytes, intestinal epithelium, skeletal muscle cells, and possibly astrocytes [1,26]. In humans, resistin is synthesized primarily in monocytes and macrophages, particularly in visceral adipose tissue, which has a high metabolic turnover [27].

Although resistin was first described as a factor contributing to insulin resistance and diabetes mellitus in humans, it has also been linked to obesity, although its exact role remains unclear [25]. Research suggests that resistin may modulate molecular signaling pathways involved in metabolic, inflammatory, autoimmune, and cardiovascular diseases [28-30]. Recent studies suggest that resistin may directly cause endothelial dysfunction and has been associated with coronary heart disease and cardiovascular disease (CVD)-related mortality [31-33]. Despite some controversy, several studies suggest that resistin plays a central role as a driver of several metabolic pathways, such as angiogenesis, thrombosis, and vascular smooth muscle cell (VSMC) migration and proliferation, all of which contribute to atherosclerosis [34].

## Review

Obesity is considered a risk factor for the development of certain cancers and epidemiological studies suggest a link between resistin and cancer, as serum resistin levels are elevated in several cancers, including breast and colorectal [35-38]. One way that resistin contributes to cancer is through inflammatory pathways since resistin is highly expressed in immune cells that infiltrate adipose tissue, and its levels are an indicator of a person's inflammatory status [39]. This suggests that resistin may influence cancer development and progression by linking obesity to an increased inflammatory state, thus contributing to tumor development [3]. Studies have shown that resistin stimulates the production of proinflammatory cytokines such as TNF-α and IL-12, which are mediated by the transcription factor NF-κB and promote inflammation [40-42]. This creates a cycle that amplifies the carcinogenic effects of chronic inflammation via activation of NF-κB and induces proinflammatory cytokine genes such as IL-12, IL-6, and TNF-α [43]. IL-6 in turn activates pathways that stimulate cell proliferation, differentiation, metastasis, and upregulation of antiapoptotic and angiogenic proteins in tumor cells [40]. TNFα, which is also promoted by resistin, is involved in angiogenesis, metastasis, growth, and differentiation of cancer [44].

**Figure 1:**
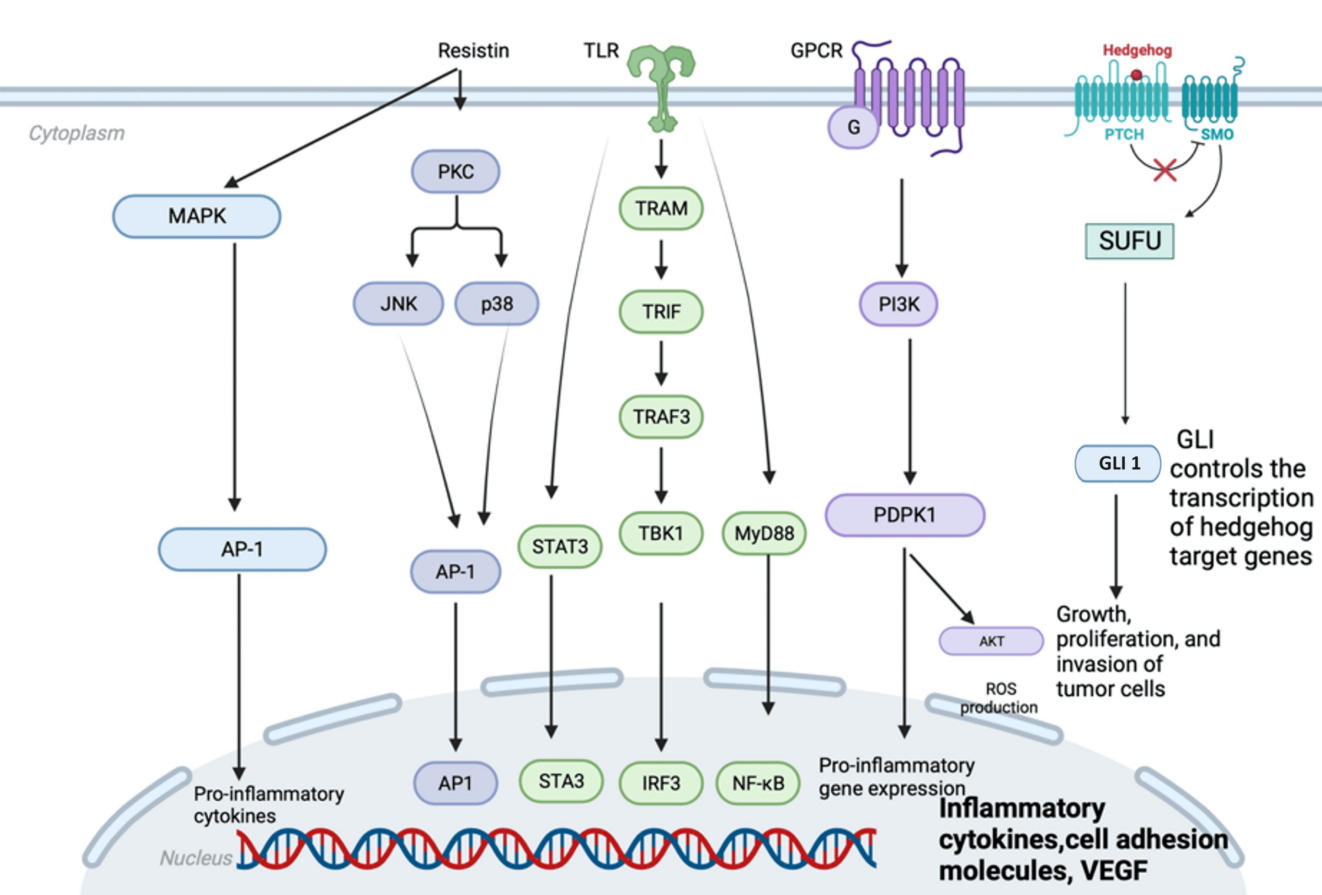
Resistin signaling pathways. Resistin binding to the TLR4, GPCR, and unknown receptors activates several signaling pathways leading to the immune cell activation and migration, neutrophil dysfunction, proinflammatory cytokines and chemokines production, and NETs formation. The image is created by the author (Rompou AV) of this study. NETs: neutrophil extracellular traps; VEGF: vascular endothelial growth factor

Resistin interferes with several mechanisms involved in carcinogenesis, including direct activation of cancer cells through stimulation of TLR4 receptors (Figure 1) [45]. Toll-like receptors (TLRs) are a class of proteins that play an important role in the innate immune system [46]. TLRs recognize invading pathogens as well as endogenous danger molecules derived from dying cells and damaged tissues and play a key role in linking innate and adaptive immunity [45,47,48]. TLR4 has been shown to serve as a receptor for the proinflammatory effects of resistin in human cells, and resistin thus promotes the progression of carcinogenesis via inflammatory pathways. This may also explain the multifaceted role of resistin in chronic inflammation, atherosclerosis, and insulin resistance [49-51].

Resistin also contributes to cancer progression by stimulating cell proliferation through activation of the PI3K and MAPK pathways (Figure 1) [52,53]. It activates transcription factors such as NF-κB that further promote the initiation, promotion, and progression of carcinogenesis [54,55]. The role of resistin in cancer spread is facilitated by promoting cell adhesion molecules that help in cell translocation, a crucial step in metastasis [44,56]. In addition, resistin promotes tumor angiogenesis and progression by inducing vascular endothelial growth factor (VEGF) expression and increasing the expression of matrix metalloproteases, which contribute to tumor vascularization, cell invasion, and metastasis [42,57].

The Sonic Hedgehog (SHH) signaling pathway is crucial for regenerating and differentiating epithelial cells in the adult colon [58]. SHH ligands, produced in the endoplasmic reticulum, are released through the dispatch membrane protein, initiating a paracrine signaling cascade. Upon binding to Patched (Ptc) protein in neighboring cells, SHH inhibits Ptc function, allowing activation of Smoothened (Smo) protein. Smo, located in the primary cilia of the intestine, regulates intracellular GLI proteins, particularly releasing GLI2 from a suppressor complex and activating it as a transcriptional activator. GLI2 then phosphorylates GLI1, which translocates to the nucleus and activates SHH-target genes. Additionally, Smo inhibits GLI3, a transcriptional inhibitor. In CRC tissues, elevated levels of SHH, Smo, and GLI1 are observed [58]. Studies show that speckle-type POZ protein (SPOP) reduces tumor growth and increases apoptosis in CRC, partly by ubiquitinating and degrading GLI2 [59]. Dysregulation of the pathway, via Ptc or Smo mutations, can contribute to CRC development [59]. The Hh signaling pathway plays an important role in several cancers, including CRC [59-61]. Resistin enhances Hh signaling pathway activity in pancreatic cancer cells, promoting cancer cell growth and survival [61]. It is also involved in the regulation of the Hh pathway in the context of adipose tissue and obesity, suggesting a role in adipogenesis and adipocyte function.

The association of resistin with inflammatory status is well documented. During inflammation, reactive oxygen species (ROS) and nitrogen species (RNS) are formed in inflammatory and epithelial cells, damaging a variety of biomolecules such as nucleic acids, proteins, and lipids [62]. ROS and RNS can damage DNA and provoke mutagenic lesions such as 8-oxo-7,8-dihydro-2′-deoxyguanosine (8-oxodG) and 8-nitroguanine [63]. In particular, the formation of 8-nitroguanine may act as a key molecular event for various types of inflammation-related carcinogenesis [63,64]. ROS induces carcinogenesis by initiating and promoting cell proliferation, apoptosis resistance, neovascularization, invasion, and metastasis [65].

Resistin and colorectal cancer

Colorectal cancer (CRC) is the fourth most common cancer worldwide. In 2018, approximately 576,000 new cases were recorded in males and 521,000 in females [66-68]. The role of obesity and physical inactivity in the development of CRC is widely recognized, and recent studies have shown that CRC patients also have metabolic alterations, such as hyperinsulinemia and insulin resistance [69,70]. Because of the impact of obesity on the development of CRC and the role of resistin in insulin resistance, research has focused extensively on measuring resistin levels in CRC patients. Interestingly, several analyses have concluded that elevated resistin levels as well as other adiponectins are significantly associated with the development of CRC (Table 1) [3,4,71-77]. Serum resistin concentrations have not been shown to be related to the degree of dysplasia, histologic differentiation, or tumor location, but have been shown to increase gradually with tumor stage progression [74-77]. Moreover, resistin concentrations are not significantly related to body mass index (BMI) values, polyp localization, or vascular invasion [3,74,76]. However, higher concentrations of resistin are observed in CRC tissues [78].

**Table 1:** Association of resistin, leptin, adiponectin, and visfatin with the development of colorectal cancer. The table indicates that resistin levels are consistently higher in CRC patients, with statistically significant p-values. Leptin levels are generally lower in CRC patients, though results are often not significant. Adiponectin and visfatin show mixed results, with some studies reporting significant differences while others do not. CRC: colorectal cancer; n.e.: not evaluated; n.s.: not significant

Biomarker	CRC versus control patients	p-Values	Reference
Resistin	Higher in patients	P<0.0001	[73]
Leptin	Lower in patients	P=n.s.	
Adiponectin	n.e.	-	-
Visfatin	n.e.	-	-
Resistin	Higher in patients	P<0.001	[74]
Leptin	Lower in patients	P=0.01	
Adiponectin	n.e.	-	-
Visfatin	n.e.	-	-
Resistin	Higher in patients	P<0.01	[75]
Leptin	Lower in patients	P=n.s.	
Adiponectin	Lower in patients	P=n.s.	
Visfatin	Higher in patients	P<0.01	
Resistin	Higher in patients	P<0.05	[76]
Leptin	Lower in patients	P<0.05	
Adiponectin	Lower in patients	P<0.05	
Visfatin	n.e.	-	-
Resistin	Higher in patients	P<0.0001	[76]
Leptin	n.e.	-	
Adiponectin	Lower in patients	P=0.03	
Visfatin	n.e.	-	-
Resistin	No significant alteration	P=n.s.	[80]
Leptin	n.e.	-	
Adiponectin	Higher in patients	P<0.05	
Visfatin	n.e.	-	-
Resistin	No significant alteration	P=n.s.	[81]
Leptin	Lower in patients	P<0.01	
Adiponectin	Higher in patients	P=n.s.	
Visfatin	n.e.	-	-

On the other hand, several research analyses have refuted the suggestion that resistin levels are associated with CRC risk (Table 1) [79-81]. A previous study involving males over 31 years concluded that serum resistin levels cannot be considered a risk factor for the development of CRC or early colorectal dysplasia. In addition, no significant association between resistin and colorectal malignancy was found when postmenopausal females were studied in relation to CRC [80,81]. Another analysis also showed no significant association between serum resistin and the development of CRC. However, when the corresponding patients were divided into rectal and CRC, resistin was positively associated with tumor size and tumor grade, but only in patients diagnosed with rectal cancer, with a higher trend observed specifically in the male group [81]. Interestingly, no significant association was found in the females studied [81].

Conflicting results emerged when resistin levels were measured in patients with colorectal adenomatous polyps. In particular, previous analysis by Kumor et al. suggested that serum resistin levels were significantly elevated in patients diagnosed with colorectal adenomatous polyps compared with the polyp-free cohort [76]. Of note, these patients were selected as cases with normal glucose metabolism [76]. In contrast, a recent study indicated that resistin levels showed no significant difference between patients with colon polyps and healthy individuals (polyp-free cases). However, significantly higher resistin concentrations were observed in patients diagnosed with colon polyps and prediabetes than in healthy individuals (polyp-free with normal glucose tolerance). These results suggest that the status of glucose metabolism in the patients studied may play a fundamental role in the association of resistin with precancerous colorectal lesions [82].

Resistin and interaction with other factors in colorectal cancer

Several factors are thought to interact with resistin promoting the development of colorectal tumors. For example, a positive correlation has been observed between high resistin and Fascin-1 expression in CRC tissues, suggesting that prognosis is worse when both proteins are elevated compared to conditions with elevation in the level of only one protein [78]. In addition, measurement of plasma resistin levels in combination with resistin mRNA and CAP-1 mRNA (adenylate cyclase-associated protein 1; resistin receptor) in peripheral blood mononuclear cells (PBMCs) has independently shown significant predictive value for CRC risk. CRC samples exhibited increased plasma levels of resistin, along with overexpression of CAP -1 mRNA and downregulation of resistin mRNA in PBMC. Moreover, a negative correlation was found between plasma resistin levels and HDL-C cholesterol in CRC patients, suggesting that HDL-C cholesterol may be involved in the modifications of the secretion process of resistin in CRC [83].

Resistin is also involved in predisposing patients to CRC via the insulin pathway [1,22,77]. Significantly higher levels of resistin and the Homeostasis Model Assessment of Insulin Resistance Index (HOMA-IR) were found in CRC patients compared with healthy subjects. Resistin was positively associated with insulin levels in CRC, whereas blockade of resistin by antibodies resulted in a decrease in blood glucose levels and insulin resistance [22]. It is concluded that glucose metabolism status along with resistin should be considered in risk assessment for the development of precancerous colorectal lesions and colorectal carcinomas. The glucose metabolism situation of patients could explain the conflicting results observed in different studies. However, further analyses are needed to support these hypotheses.

Genetic variability of resistin gene in colorectal cancer

The human resistin gene (RETN), located on chromosome 19p13.2, consists of four exons that produce a protein of 108 amino acids [84]. The protein is a 10-kDa cysteine-rich polypeptide that contains an 18-amino acid signal region and a 90-amino acid mature region. Structural analysis revealed five intramolecular disulfide bonds and multiple β-turns [44,85]. Resistin is known to form oligomers that circulate in human serum as homotrimers, homohexamers, or higher molecular weight oligomers. The lower molecular weight isoforms are considered more active [85,86].

In recent years, the study of RETN gene polymorphisms has gained popularity since Cho et al. reported that the nucleotide variation C420G (rs1862513), located in the promoter of the RETN gene, plays a central role in RETN gene regulation [87]. Notably, significantly high levels of resistin have been detected in plasma of 420G carriers [87]. Given the association of this polymorphism with resistin expression levels and its potential impact on CRC growth, numerous research analyses have focused on the prevalence of the RETN C420G polymorphism in CRC patients, with controversial results [88-92]. For example, an analysis of the RETN C420G polymorphism in patients from Saudi Arabia showed that individuals who have the heterozygous RETN 420CG genotype have an increased risk of developing CRC [88]. In contrast, data from Iranian patients show that the RETN 420CG genotype is evenly distributed between patients and the control group, whereas the RETN 420CC genotype appears to have a protective effect against the development of CRC [23]. Interestingly, no significant association between the different resistin genotypes and the risk of CRC growth was described in patients from Turkey, the Czech Republic, Sweden, and Belgrade [83,89-91]. A recent meta-analysis found that the rs1862513 (C420G) variant significantly increased CRC risk and overall cancer risk in Caucasians [92]. Considering all these data, it was concluded that further analysis in different ethnic groups and geographic regions is needed to better understand the impact of this polymorphism on CRC growth and whether this polymorphism can be used as a valuable molecular biomarker for early detection.

Although the effects of the RETN C420G polymorphism on CRC growth remain unclear, two other RETN polymorphisms (G299A, C180G) were studied in CRC patients from Saudi Arabia [88,93]. Notably, the heterozygous RETN 299GA genotype was found to increase the susceptibility of Saudi patients to develop CRC [88], whereas no significant association was found between the RETN C189G polymorphism and CRC risk [93]. Nevertheless, there is no information on the prevalence of these two polymorphisms in CRC patients from different geographic regions, and no other polymorphisms of the RETN gene have been studied. Therefore, comprehensive sequence analysis of the RETN gene in CRC patients is crucial to clarify whether the polymorphic variability of the RETN gene may influence the susceptibility of patients to develop colorectal neoplasia.

Resistin and chemotherapy in colorectal cancer

The correlation between levels of various adipokines and chemotherapy was investigated in two recent studies by the same research team that focused on CRC [70,94]. In the first study, 34 patients who had undergone surgical resection and had metastatic disease, were treated six times with 5-fluorouracil (5-FU) based chemotherapy. Patients were evaluated using Response Evaluation Criteria in Solid Tumors (RECICTν.1.1) based on radiological findings and CEA values. The research results indicated that resistin and visfatin levels had significantly decreased after cancer treatment. In addition, a significant decrease in insulin levels was also observed. In contrast to resistin and visfatin, average leptin levels increased, while adiponectin levels also increased by 47% [70].

In the second study, 42 patients with advanced colorectal cancer (CRC) undergoing palliative treatment were evaluated [94]. Plasma levels of adipokines were assessed using ELISA before and after six cycles of chemotherapy. The findings indicated a significant reduction in resistin and visfatin levels following 5-FU-based chemotherapy among patients showing partial response (PR) or stabilizing disease (SD), while those with progressive disease (PD) did not exhibit such changes. Leptin levels increased significantly in PR and SD subjects by 19% and 21%, respectively, with no significant changes observed in PD patients relative to baseline values. Moreover, significant differences in leptin levels between patients with PR versus PD or SD versus PD persisted after chemotherapy.

Adiponectin levels did not significantly differ between PR, SD, and PD subjects before chemotherapy. Following six courses of treatment, adiponectin levels in PR patients increased by 13%, whereas no changes were observed in the SD or PD groups following chemotherapy. These results underscore chemotherapy's potential to elevate anti-inflammatory adiponectin levels and reduce visfatin and resistin levels. The study proposes that changes in leptin, visfatin, and resistin could serve as predictive biomarkers for chemotherapy response, highlighting their clinical relevance in assessing treatment outcomes [94].

Finally, a recent study found an association between levels of resistin-like molecules (RELM), a family of recently identified proteins that play a role in various disease contexts, CRC, and disease progression [21,95,96]. These molecules are involved in inflammatory processes with microbial etiology, inflammatory diseases, and metabolic disorders, and some members of this family are associated with tumor progression [97]. This study showed significant differences in colon resistin-like beta (RETNLB) transcript during the progression of CRC, suggesting that resistin may be a potential novel target protein for prognosis in CRC patients. In addition, adjuvant therapy can reduce RETNLB, suggesting that this protein has the potential to act as a therapeutic target in CRC patients [21]. Resistin antagonism may be a potential strategy to counteract resistance to chemotherapy [98]. Further studies in vivo need to be performed with other chemotherapeutic agents used in the treatment of CRC to determine the signaling pathways involved and ways to overcome resistance to therapy.

Discussion

Obesity is a major global epidemic, and identifying the mechanisms underlying obesity-induced cancers is critical. Obesity induces dysregulated secretion of resistin, which is closely related to tumorigenesis in CRC, mainly via an inflammatory cascade. There are clear interactions between resistin and other adipocytokines, all of which are affected by lipid and glucose metabolism. Further studies should compare genetic variations and levels of adipocytokines, lipids, and glucose to investigate the underlying mechanisms. These findings may optimize our approach to prevention and treatment of CRC. The role of resistin in CRC is not fully understood, but several mechanisms have been proposed to understand its involvement in cancer. The primary secretion of resistin by adipose tissue and its role in inflammation have been linked to the promotion of chronic inflammation, which is a known risk factor for cancer [39,64,99].

Studies have shown that resistin can directly affect the growth and proliferation of colon cancer cells. It has been shown to promote cancer cell survival and growth by activating signaling pathways involved in cell survival, such as the PI3K/Akt and NF-κB pathways. In addition, resistin can promote angiogenesis by inducing the expression of vascular endothelial growth factor (VEGF) and increasing the expression of matrix metalloproteases, which contribute to tumor vascularization and promote cell invasion and metastasis [100,101]. Insulin resistance may be another way that resistin interferes with tumorigenesis because resistin contributes to insulin resistance by interfering with insulin signaling pathways. Insulin resistance may create an environment conducive to tumor growth and survival [51,77].

Clinical studies have provided evidence of a possible association between resistin concentrations and CRC. Elevated resistin concentrations have been observed in the serum or tumor tissue of CRC patients compared with healthy individuals [74]. In addition, higher resistin concentrations have also been associated with more advanced stages of CRC and poorer prognosis [102]. Resistin appears to decrease in patients after the administration of chemotherapeutic agents and therefore may play a role as a prognostic molecule. Given the emerging link between resistin and CRC, targeting resistin or its downstream signaling pathways may have therapeutic potential. Inhibition of resistin could potentially reduce tumor growth, attenuate inflammation, and improve insulin sensitivity in CRC. However, further research is needed to understand the exact mechanisms and explore therapeutic possibilities.

In summary, resistin appears to play a role in the development and progression of CRC, possibly through its involvement in inflammation, tumor growth, and insulin resistance. Understanding the mechanisms underlying the link between resistin and CRC may offer insights into new therapeutic strategies for this disease. Further research is needed to elucidate the precise mechanisms and evaluate the clinical significance of resistin in CRC.

## Conclusions

Resistin is an adipokine recognized as a key factor in the pathogenesis of colorectal cancer. Initially linked to insulin resistance and inflammation, high levels of resistin have been associated with various obesity-related diseases, including diabetes, cardiovascular disease, and several cancers. Resistin's role in colorectal cancer appears multifaceted, promoting tumorigenesis through chronic inflammation, insulin resistance, and direct effects on cancer cell proliferation and survival. It activates key signaling pathways such as PI3K/Akt and NF-κB and stimulates proinflammatory cytokines like TNF-α and IL-6, facilitating tumor growth, angiogenesis, and metastasis. Elevated resistin levels have been observed in colorectal cancer patients, correlating with advanced tumor stages and poorer prognosis. Additionally, specific polymorphisms in the resistin gene have been linked to colorectal cancer risk, though findings are inconsistent across different populations. Chemotherapy has been shown to reduce resistin levels, indicating its potential as a prognostic marker. Targeting resistin and its pathways may offer novel therapeutic strategies for colorectal cancer, but further research is essential to understand its mechanisms and clinical applications fully.
